# Retrospective evaluation of udder recovery of cows with subclinical mastitis following treatment with acoustic pulse technology (APT) on commercial dairy farms and its economic impact

**DOI:** 10.1371/journal.pone.0303947

**Published:** 2024-05-31

**Authors:** Uzi Merin, Dani Gilad, Shamay Jacoby, Benny Keynan, Yochai Hefer, Yaniv Lavon, Gabriel Leitner

**Affiliations:** 1 Gedera, Israel; 2 Armenta Ltd., Ra’anana, Israel; 3 Institute of Animal Science, Agricultural Research Organization, The Volcani Center, Bet Dagan, Israel; 4 Hafer Dairy Farm, Haogen, Israel; 5 Nofim Dairy Farm, Gazit, Israel; 6 Israel Cattle Breeders Association, Caesarea, Israel; University of Illinois, UNITED STATES

## Abstract

Retrospective evaluation of udder recovery following treatment of the inflamed quarter with acoustic pulse technology (APT) of cows with subclinical mastitis was done on 4 Israeli commercial dairy farms. Here, we evaluated the APT treatment as a tool to manage subclinical mastitis and its economic consequences in commercial farms. Recovery of the infected glands following APT treatment was compared to the customary no-treatment (NT) for cows with subclinical mastitis. Over 2 years, 467 cows with subclinical mastitis were identified. Subclinical mastitis was defined by elevated somatic cell count (SCC; >1 × 10^6^ cells/mL) in the monthly test-day milk sample; 222 cows were treated with APT and 245 cows were not treated and served as control. Differences between treatment groups in culling, milk quality, milk yield and bacterial elimination were analyzed. After treatment, cure from bacteria was calculated only for cows with pre-isolated bacteria. The percentage of sampled cows determined as cured (no bacterial finding) in the NT group was 32.7% (35/107) (30.9% Gram negative; 32.4% Gram positive) and in the APT-treated group, 83.9% (42/55) (89.4% Gram negative; 80.6% Gram positive). Culling rate due to mastitis was significantly lower (>90%) in the APT-treated vs. NT group. Recovery was 66.0% in the APT group compared to 11.5% in the NT group at 90 d post-treatment. Average milk volume per cow in the APT-treated group was 16.1% higher compared to NT cows. Based on the study, savings incurred by using APT to treat only subclinical cows per 100-cow herd can total $15,106/y, or $309 per treated subclinically infected cow.

## Introduction

Mastitis, defined as inflammation of the mammary gland, results from several factors. Causative infecting agents can be viruses, mycoplasmas, or fungi, but most are bacteria. Damage to the tissue due to the inflammation process may persist long after elimination of the infection caused by the pathogen [1, 2].

Mastitis can be clinical or subclinical, depending on the type and presentation of symptoms. Clinical mastitis is visually detected, while subclinical mastitis can only be specifically diagnosed by measures such as somatic cell count (SCC), California mastitis test (CMT) and/or bacteriological tests [1, 3]. Use of antibiotics as a preventive or treatment measure for clinical mastitis is the most common practice [4–6]. Antibiotics are used for more than half a century, however, the prevalence of mastitis has not changed over the years, as new mastitis pathogens developed and the rate of cows that require therapy remains the same [7]. Antibiotics are becoming less readily available to farmers due to consumers’ growing awareness of animal welfare and the spread of bacteria with antimicrobial resistance (**AMR**), pushing decision-makers to reduce antibiotic use in livestock production [8, 9]. Thus, any mastitis treatment should be evidence-based to encourage the justified use of antibiotics [10], resulting in a growing gap between the demand for a solution to treat mastitis and the number of therapies available on the market. Moreover, the focus of prudent use should be on preventing mastitis through improved management.

When the inflammation cannot be visually identified with clinical signs, it is defined as subclinical mastitis, even though many subclinical mastitis cases are infected with the same pathogens as in clinical cases. Thus, clinical mastitis cases are often flare-ups of underlying more long term subclinical intramammary infections. Prevalence of subclinical mastitis can range from 25–35% of the cows in a herd [11]. Some of these cows will be in a chronic stage, resulting in loss of milk production that can reach up to ∼6.5 kg/d for cows with over 250,000 SCC/mL [12, 13] and reduced milk quality [14]. Moreover, a negative influence on reproduction [15, 16], and premature forced culling [17] can be noted. Most subclinical mastitis cases are not treated during lactation, because the use of antibiotics invokes discarding the milk. However, expected spontaneous cure rates are relatively low [18] thus the economic influence is high.

Subclinical mastitis in commercial dairy herds, refers to cows whose SCC increases to levels that influence bulk milk SCC and, therefore, may affect milk value for the farmer. Thus most of those caws ignored if their milk is not expected to increase bulk tank SCC payment level cutoff and only those cows diagnosed by the routine milking test with SCC > 1 × 10^6^ cells/mL usually need the attention of the farmer [19, 20].

Therefore, new approaches of treating subclinical mastitis need to be developed, as reflected by studies aimed at decreasing the use of antibiotics in dairy production [21].

Acoustic pulses, also called low-intensity shockwaves, are “mechanical” waves characterized by an initial positive and very rapid high-amplitude phase, followed by a few microseconds, a sudden phase of mild negative pressure and then a return to the ambient (basic) values [22]. The low-intensity shock waves affect cells by mechanotransduction, where different cell types sense and process the mechanical information and convert it into biochemical responses. Mechanotransduction influences important cellular functions, such as migration, proliferation, differentiation, and apoptosis [23, 24]. As a result, arterioles are remodeled, stimulated to grow, and new ones are ultimately formed. The new blood vessels improve blood supply and oxygenation in the treated area and support faster healing. Low-intensity shockwaves also show anti-inflammatory effects [25, 26]. They modulate endogenous nitric oxide (**NO**) production under both normal and inflammatory conditions. Data on the rapid enhancement of endothelial NO synthase activity in extracorporeal shockwave-treated cells suggest that increased NO levels and the subsequent suppression of NF-κB activation may account, at least in part, for the clinically beneficial action on tissue inflammation [25]. Low-intensity shockwaves have been used for the last 20 yr in human healthcare for orthopedic treatments, physiotherapy, sports medicine and vascular and urology treatments, and in veterinary medicine for racehorses; its main intended use is to speed up tissue recovery by producing new blood vessels locally (at the treatment site), reducing inflammation and improving overall tissue functioning, alongside other positive long-term effects [25, 27].

In acoustic pulse technology (**APT**), acoustic pulses are generated by repeatedly driving a projectile using high air pressure to collide against an anvil that is connected to a treatment head that is in contact with the body [28]. The impact produced by the collision is converted into an acoustic pulse, which is then transferred noninvasively to the target areas of the animal to be treated. Specific development of this treatment for dairy cows, which produces mechanical waves at therapeutic levels distributed over a large area, has made it more practical for treating the cow’s udder than other existing technologies [28–30]. The volume of the treatment zone is estimated to cover around 8,500 cc^2^ of the udder tissue, deeper and larger than that of any other technology existing today in human healthcare [1].

The APT-X pulse generator (Armenta Ltd., Ra’anana, Israel) is the first device to be based on the principles of APT and specifically designed to address the unique needs in treating cow udders on a dairy farm. The APT triggers the cow’s self-healing mechanism and can be used regardless of the cause of infection, thus negating the need to identify the causative pathogen [24, 28]. The APT-X is a handheld device that allows easy access to the treatment site—the infected quarter—and it is connected to a compressed air tank, allowing greater mobility, without the need for electrical power. The treatment is harmless to the animal, with no side effects [28]. Furthermore, it does not affect the milking routine because there is no need to discard milk from the uninfected glands, as must be done when antibiotics are used. The technology is environmentally friendly with reusable parts [27]. In previous studies [29, 30], APT was assessed on hundreds of dairy cows with clinical or subclinical mastitis and its use resulted in ∼70% recovery compared to only ∼20% recovery in a nontreated control group. Moreover, among the treated cows with identified bacteria, 52.6% of the glands were cured compared to only 25.0% in the control group.

The evaluated APT treatment as a tool to manage subclinical mastitis and its economic consequences for commercial farms was studied. The retrospective analysis evaluated udder recovery in cows with subclinical mastitis following treatment with APT for 2 yr on 4 dairy farms, as identified by monthly SCC. The analysis included cows with first SCC elevation of >1 × 10^6^ cells/mL at the test-day milk recording, that were treated by APT or not treated (NT), the latter serving as a control group. The analysis compared recovery from inflammation, culling, and milk yield (**MY**).

## Materials and methods

### Study layout

Data for the retrospective analysis were collected from 4 dairy herds where the APT-X had been used routinely for 2 yr: a commercial herd of 700 lactating Israeli Holstein cows (Hafer dairy farm, Kibbutz Haogen, Israel) (A); a herd of 210 Israeli Holstein cows at the Agricultural Research Organization (ARO) dairy farm, Bet Dagan, Israel (B); a commercial herd of 1,200 lactating Israeli Holstein cows (Haemek dairy farm, Kibbutz Yifat, Israel) (C) and a commercial herd of 700 lactating Israeli Holstein cows (Nofim dairy farm, Kibbutz Gazit, Israel) (D). The four Israeli Holstein dairy farms are typical to Israel, representing large, medium and small sized herds. The dairy parlors were equipped with an online computerized dairy management system controlled by AfiFarm Herd management software (Afimilk, Kibbutz Afikim, Israel) (http://www.afimilk.com) or SCR Dairy Cow Monitoring and Herd Management systems (MSD, Netanya, Israel) (http://www.scrdairy.com). The cows were milked thrice daily with MY of 11,900–12,850 L for 305 d of lactation. The cows were kept in an open shed padded with composted daily cultivated bedding. They were fed a total mixed ration (TMR) containing 16.8% crude protein and 1.75 Mcal/kg dry matter. The health status in these herds was good with ∼15/100 cows/year showing clinical mastitis, mostly caused by Gram negative bacteria, 20–25% subclinical mastitis caused by coagulase-negative staphylococci (CNS) and with 190–220 × 10^3^ SCC in the bulk milk tank. During period of 2017–2019, cows identified with subclinical mastitis at the routine monthly milk test—showing a first-time elevation of SCC >1 × 10^6^ cells/mL as recorded in the Herd Book of the Israeli Cattle Breeders Association (ICBA, Caesarea, Israel), were enrolled for the analysis.

Because no visible clinical symptoms were noted, cows were considered as having subclinical mastitis. After each test day, the farm received the results through ICBA’s herd management software (Israeli dairy farm management program ‐ NOA). The infected quarters were identified and validated using CMT using the scale 0, T (trace). 1,2,3, as was suggested (https://extension.missouri.edu/publications/g3653). Only positive CMT (≥1) infected quarters and no more than 2 inflamed quarters/cow were selected to the study. Cows were selected randomly once a month following the routine monthly milk test, and were divided according to lactation number, day in milk, level of CMT and number of inflamed quarter/cows. Bacteriological status was not a parameter for designation. 222 cows were treated by APT (APT-X) and 245 were NT and served as a control. Only 3 cows in each group had 2 inflamed quarters. A cow that was pretreated with antibiotics was excluded from the study.

Following CMT, sterile samples were taken from all the cows included in the study, only from the CMT-positive quarters for bacteriology testing at the bacteriological laboratory of the Israeli Dairy Board (Caesarea, Israel). Specific details for the bacteriology testing can be found in Leitner et al. [1]. Briefly, 10 μL from each sample was spread on blood agar plate and on MacConkey agar plates and incubated for 24–48 h at 37°C. Bacteria were identified by classical bacteriological methods.

Twenty to thirty days after treatment, only cow that still had CMT (≥1) in the infected quarters were resampled for bacteriology. Only in farm C and D, if a quarter was found positive, a second sample was taken for bacteriology. Therefore, the bacteriological analysis was performed only for farms C and D. Twenty to thirty days after treatment, only cow that still had CMT ≥1 in the infected quarters were resampled for bacteriology. Cows were considered cured if the bacteria found in the milk sample before APT treatment or in the NT group were not found in the milk sample after treatment in the same quarter. If a quarter was found negative for CMT it was also assumed negative for bacteria.

The APT protocol consisted of 400 pulses delivered on the surface of the infected quarter on 2 sides of the teat (200 pulses each). Each course of treatment consisted of 3 daily treatments (each lasting 3 min), given for 3 d, with a 2- to 3-d interval between treatment days, for a total of 3 × 3 min treatments weekly [24, 26].

Data for the analyses included lactation number, days in milk (DIM), daily milk yield (MY), SCC at time of treatment, SCC and daily MY of the 2 previous monthly test days and up to 3 monthly test days after the treatment. Management decisions regarding dry-off of the infected gland and culling of the cows were also recorded. Milk samples from the infected glands were taken by farm personnel for bacteriological analysis.

Recovery from inflammation was defined as a decrease in SCC < 250 × 10^3^ SCC/mL, or and <500 × 10^3^ cells/mL to in at least two of the three monthly milk recordings after treatment. Recovery was calculated according to SCC level up to 3 months post-treatment. 1. According to 2 cutoff levels: <250 × 10^3^ and <500 × 10^3^ cells/mL, where cows with lower levels of SCC than those cutoff levels in 2 out of 3 test-day milk samples post-treatment were considered recovered, and 2. When reduction in SCC of each individual cow from the baseline (day of treatment) on each of the 3 monthly test days after treatment was >75%. The use of the 2-cutoff levels are being used due to practical reasons. Scientists consider <100, or 200 × 103 SCC/mL as healthy or clinically recovered, while, for many farmers < 500 × 103 level is practically acceptable.

### Statistical analysis

Prior to the beginning of the experiment, different factors that can affect cow response to the treatment were analyzed using T-test. Data on recovery (decrease in SCC level to <250 × 10^3^ or <500 × 10^3^ cells/mL on 2 out of 3 milk test days after treatment or reduction of more than 75% from the SCC baseline) were tested by a multivariable model designed with a logistic model statement using the GLIMMIX procedure of SAS (version 9.2, SAS Institute, Cary, NC), with recovery results as the dependent variable. In the first model, we included the herd (4 different dairy farms) in the model, but because there was no interaction between farms, we ran the final model without the herd variable: % recovery = intercept + parity + treat + parity × treat + error, where % recovery = ln P/(1−P), with P being the probability of recovery; parity = 1^st^, 2^nd^, or 3^rd^ and more lactations; treat = the 2 different treatments (APT, NT) given to the cows. All variables were considered as fixed effects. The dependent variables were the percentage of recovered cows according to the 2 SCC cutoff levels (<250 × 10^3^ or <500 × 10^3^ cells/mL) for the different treatments, and levels of >75% reduction in SCC from baseline on day of treatment in milk samples from 3 monthly test days post-treatment. Bonferroni analysis was performed for each treatment to determine differences between them. Adjusted odds ratio (**OR**) and 95% confidence intervals (**CI**) are reported.

Differences in the percentage of culled cows until 90 d post-treatment for mastitis were analyzed using the GLIMMIX procedure of SAS (version 9.2) with the general form: % culling = parity + treat + parity × treat + error, where parity = 1^st^, 2^nd^, or 3^rd^ and more lactations, treat = the 2 different treatments (APT, NT) given to the cows. To normalize the SCC, data were converted to somatic cell score (**SCS**) using Log10. For the analysis of MY and SCS, the statistical model included effects of treatment (APT, NT), cows (within treatment), days from infection and treatment-by-time interaction.

The MY calculation was performed twice:

1. Individual MY: This analysis was performed on all cows that were treated and monitored for 3 monthly test days after treatment. Time of treatment (time 0) for each individual cow was considered 100% and that on the next test day was expressed as Δ% of the volume at time 0. Cows that were culled due to the mastitis event were considered in the calculation as contributing 0 L after the culling date.
2. Total volume milked into the bulk milk tank: Total milk in the bulk tank of all cows in a group at time of treatment (time 0) was set at 100% and that on the next milk test day was expressed as Δ% of the volume at time 0. Cows that were culled due to the mastitis event were considered in the calculation as contributing 0 L milk.

Results are expressed as the average of all cows in a group at each time point. Both analyses were carried out using the Proc Mixed procedure of SAS and had the general form: milk% at 30, 60, and 90 d post-IMI = treat + lact + treat × lact + error, with treat = 2 different treatment groups (APT, NT), parity = 1^st^, 2^nd^, or 3^rd^ and more lactations, and their interaction.

Cured percentage according to treatment group was analyzed by a model designed with a logistic model statement using the GLIMMIX procedure of SAS (version 9.2), with cured percentage as the dependent variable. % cured = intercept + treat + error. Parity and parity x treat interactions were found to be non-significant and were therefore excluded from the final model. Correlations between recovery and cure were tested in Excel. Data are presented as mean and standard error of the mean (SEM).

## Results

In the 4 herds, 467 cows were identified with first elevation of SCC >1 × 10^6^ cells/mL on the routine monthly milk test day: 174 cows in herd A, 28 in herd B, 205 in herd C and 60 in herd D; 222 cows were assigned to the APT treatment, and 245 were not treated (NT group) and served as a control.

None of the following risk factors differed among APT-treated and NT cows: MY (39.39 ± 0.7 and 40.3 ± 0.7 kg/d, respectively; P = 0.364), SCC (×10^3^) on day 0 (2,694 ± 144 and 3,059 ± 182, respectively; P = 0.348), DIM (133 ± 7.1 and 120 ± 5.6, respectively; P = 0.158) and lactation number (2.88 ± 0.1 and 3.3 ± 0.1, respectively: P = 0.179). No adverse reactions were observed during the APT sessions, no inflammatory reaction was noted on the day of treatment or the day after and milk production remained stable.

### Bacteriology

Bacteriological analysis was performed for farm C and D and the data are presented in Table 1. Of the cows, 92.1% (244/265) were sampled before treatment, with 162 cows in the NT group and 82 cows in the APT group. The distribution of the bacteria before treatment was different for the 2 treatment groups, with ∼20% (37/162) Gram positive in the NT group and 37.8% (31/82) in the APT group, and 42% (68/162) Gram negative in the NT group, and 23.2% (19/82) in the APT group. Although CMT was positive in 55 of the 162 sampled cows in the NT group (34%), there was no bacterial finding (**NBF**). Of the 82 APT-treated cows, 32 (39.0%) were NBF. Only cows that exhibited inflammation in the same quarter as on the pretreatment day by positive CMT were sampled. Of the 244 cows presenting with mastitis, 120 (49.1%) were eligible for sampling: 108 (66.6%) of the NT cows and 12 (20%) of the APT-treated cows. In the remaining 123 (50.9%), no inflammation of the udder was observed, and they were therefore not sampled and were considered as cured.

**Table 1:** Pre- and post-sampling of milk from cows for farm C and D with subclinical mastitis (CMT+) and the cure rate following acoustic pulse technology (APT) or no treatment (NT).

Group	Bact. species	Pre-treatment	Post treatment						Total cure of bacteria (%)^2^
			CMT		Bacterial finding (%)				
		Bacterial & CMT+	-	+	Positive (Same bact.)	New pathogen	Total positive	Cure (%)^1^	
**NT**	*E*. *coli*	46	12	34	19 (41.3)	11 (23.9)	30 (65.2)	58.7	30.9 (21/68)
	*Proteus*	0	0	0	0	1 (100)	1 (100)	N/A	
	*Klebsiella*	1	0	1	0	1 (100)	1 (100)	100.0	
	*Hemophilus somnus*	2	0	2	1 (50)	1 (50)	2 (100)	50.0	
	*Pseudomonas*	15	4	11	7 (46.7)	3 (20)	10 (66.7)	53.3	
	*Actino*. *pyogenes*	4	2	2	2 (50)	1 (25)	3 (75)	50.0	
	Total Gram (-)	68	18	50	29 (42.6)	18 (26.5)	47 (69.1)	57.4	
	*Strep*. *dys*.	6	0	6	4 (66.7)	1 (16.7)	5 (83.3)	33.3	32.4 (12/37)
	*Strep*. *uberis*	5	1	4	1 (20)	4 (80)	5 (100)	80.0	
	*Strep*. *spic*.	4	0	4	2 (50)	1 (25)	3 (75)	50.0	
	*Enterococcus*	13	7	6	2 (15.4)	2 (15.4)	4 (30.8)	69.2	
	CNS	9	5	4	3 (33.3)	4 (44.4)	7 (77.8)	66.6	
	**Total Gram (+)**	37	13	24	12 (32.4)	12 (32.4)	24 (64.9)	67.6	
	*Candida*	2	2	0	0	0	0	0.0	100.0 (2/2)
	Total bacteria	107	33	74	41 (38.3)	32 (29.9)	73 (68.2)	44.6	32.7 (35/107)
	NBF + positive CMT	55	11	34	N/A	7	7	**N/A**	
	**Total NT cows**	162	54	108	56	N/A	108	N/A	
**APT**	*E*. *coli*	15	13	2	0	1 (6.7)	1	93.3	89.4 (17/19)
	*Pseudomonas*	2	1	1	0	0	0	100	
	*Klebsiella*	0	0	0	0	1 (100)	1 (100)	N/A	
	*Actino*. *pyogenes*	2	2	0	0	0	0	100	
	Total Gram (-)	19	16	3	0	2 (10.5)	2 (10.5)	100.0	
	*Strep*. *spic*.	4	0	1	1 (25)	0	1 (25)	75	80.6 (25/31)
	CNS	27	17	10	3	0	3	88.9	
	Total Gram (+)	31	17	11	4 (12.9)	0	6 (19.4)	83.3	
	Total bacteria	50	24	14	4 (8)	2 (4)	8 (16)	89.4	83.9 (42/)50
	NBF+ Positive CMT	32	21	9	0	2	2	N/A	
	**Total APT cows**	82	45	23	4	2	10	N/A	

^1^ Cure = calculated for cows with the same bacteria isolate.

^2^ Total cure of bacteria = calculated for cows with no isolated bacteria (including new pathogen). Cows with no bacterial isolates before treatment were not considered cured. *E*. *coli–Escherichia coli*, *Actino*. *pyogenes–Actinomyces pyogenes*, *Strep*. *dys*.–*Streptococcus dysgalactiae*, *Strep*. *uberis–Streptococcus uberis*, *Strep*. *spic*.)*–Streptococcus spices* (species), CNS–coagulase-negative staphylococci, NBF–no bacterial finding, CMT–California mastitis test.

After treatment, cure from bacteria was calculated only for cows with pre-isolated bacteria. The percentage of sampled cows determined as cured (no bacterial finding) in the NT group was 32.7% (35/107; 30.9% Gram negative, 32.4% Gram positive), and in the APT-treated cows it was 83.9% (42/55; 89.4% Gram negative, 80.6% Gram positive) (Table 1).

The overall difference between NT and APT groups was significantly different (P < 0.001).

### Culling

**Fig 1:**
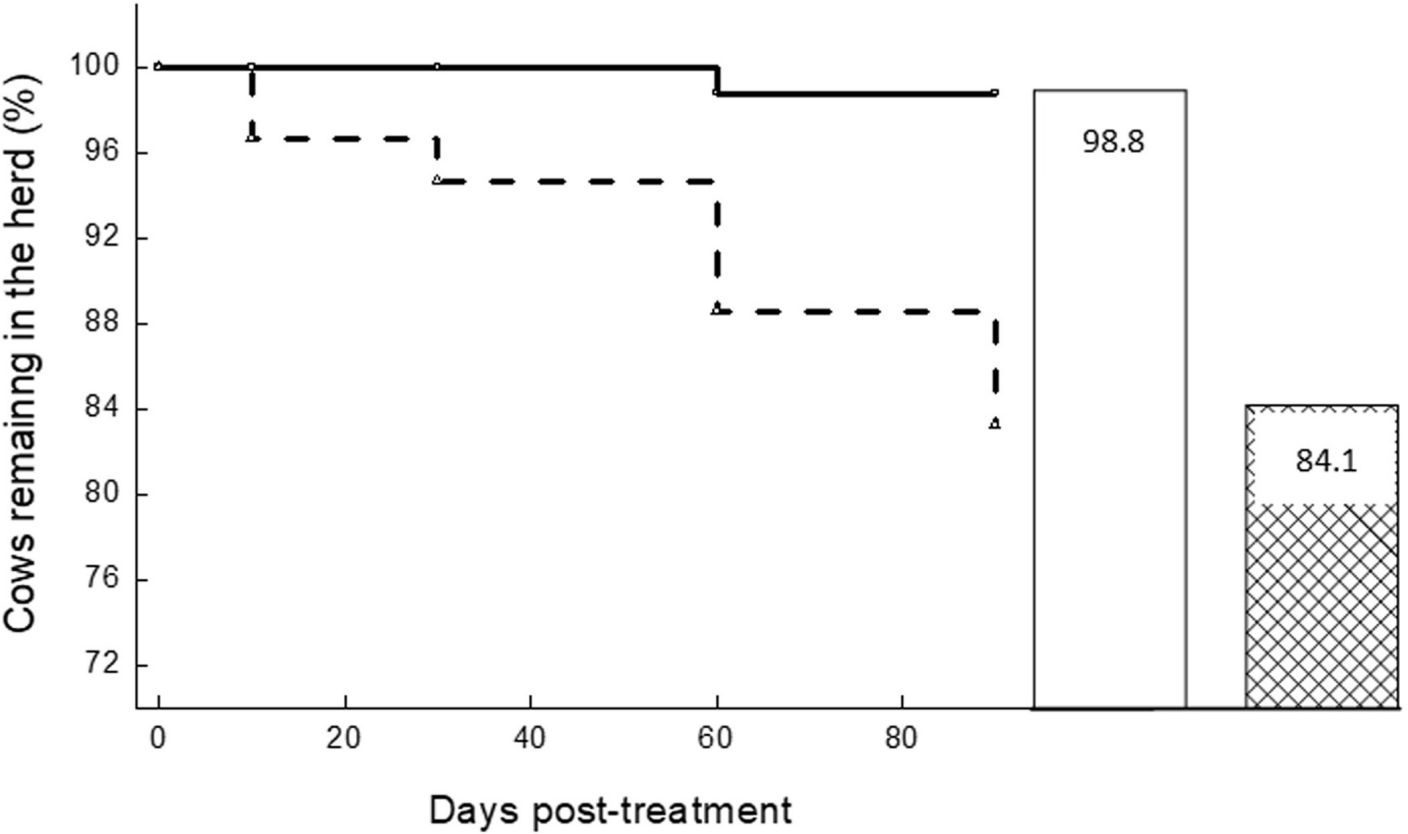
Dynamics of percent cows culled due to a subclinical mastitis event. Left: Dynamics of percent cows culled due to a subclinical mastitis event (SCC >1 × 10^6^ cell/mL) up to 90 d post-treatment; cows treated with acoustic pulse technology (APT; dotted line, n = 222) and not treated (solid line, n = 245). Right: Percentage of cows remaining in the herds on d 90 post-treatment (APT-treated, white bar; not treated, hashed bar).

During the 90 d post-treatment, a significant difference (*P* < 0.001) between groups was observed: 1.35% (3/222) cows in the APT group and 16.7% (41/245) cows in the NT group were culled due to a mastitis event (Fig 1). As a result, 98.8% of the APT-treated group remained in the herds compared to 84.1% of the NT group (*P* < 0.001; Fig 1).

### Recovery

Recovery from inflammation was defined as a decrease in SCC level of >75% (R75) and in addition, as the OR of the statistical analysis set for SCC <250 × 10^3^ (R250) and SCC <500 × 10^3^ (R500) cells/mL in at least 2 of the 3 monthly milk tests after treatment relative to the baseline on day of treatment. The OR results showed significantly higher recovery in the APT-treated group compared to the NT group (Table 2).

**Table 2:** Recovery of cows identified as subclinically infected and treated with acoustic pulse technology (APT) or not treated (NT) as a control. Odds ratio (OR) and 95% CI are presented.

		R250		R500		R75	
**Variable**	Level (n)	OR (CI)	*P*	OR (CI)	*P*	OR (CI)	*P*
**Group**	APT (222)	0.086 (0.048,0.1558)	<0.0001	0.048 (0.026,0.088)	<0.0001	0.096 (0.056,0.164)	<0.0001
	NT (245)	1		1		1	
**Parity**	1 (76)	0.330 (0.169,0.646)	0.0038	0.379 (0.196,0.733)	0.0121	0.309 (0.165,0.579)	0.0008
	2 (91)	0.577 (0.289,1.153)	0.3569	0.894 (0.439,1.821)	1	0.58 (0.314,1.073)	0.248
	3+ (300)	1		1		1	

Recovery was defined as a decrease in SCC level to <250 × 10^3^ cells/mL (R250) or 500 × 10^3^ cells/mL (R500), or a reduction of more than 75% (R75) on all the 3 monthly milk test days or on the second and third test days.

**Fig 2:**
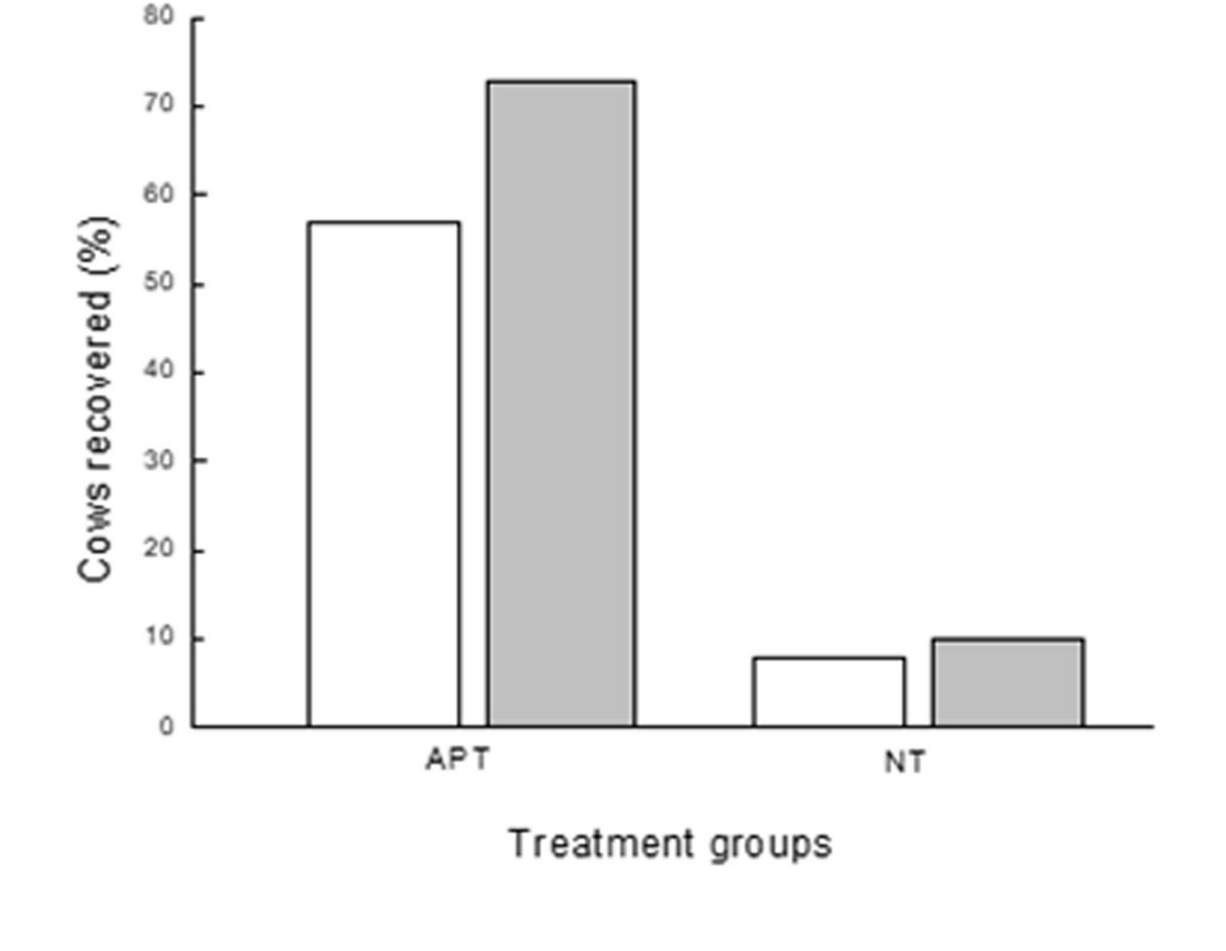
The effect of APT treatment on recovery rate. Percent cows with subclinical mastitis events (SCC >1 × 10^6^ cells/mL) recovered up to 90 d post-treatment in cows treated with acoustic pulse technology (APT n = 222) vs. no treatment (NT; gray, n = 245). Recovery = percent cows reaching SCC below 2 different cutoff levels: <250 × 10^3^ (empty bars) or <500 × 10^3^ (gray bars).

The percentage of cows with SCC levels reduced by >75% compared to baseline on each of the 3 milk test days post-treatment (30, 60, 90 d) were 54.9, 59.3, 57.4, respectively for the APT treatment and 12.2, 13.5, 11.0, respectively, for the NT group. Overall percent recovery (2 out of 3 milk test days after treatment) was 65.8% for the APT-treatment group, and 11.5% for the NT group. The calculation of recovery according to SCC up to 3 months post-treatment at the 2 different cutoff levels: <250 × 10^3^ and <500 × 10^3^ cells/Ml, is presented in Fig 2. At both SCC cutoff levels, the APT-treated group (57% and 73%, respectively) showed significantly higher recovery (*P <* 0.001; ∼7-fold) than the NT group (8% and 10%, respectively). Changing the cutoff level from <250 × 10^3^ to <500 × 10^3^ cells/mL had only a minor influence on the results. The recovery of heifers was significantly lower (*P* = 0.0008) than ≥ 2 parties (Table 2).

During that period, the number of dried-off glands (due to low milk volume, milk quality, or both) was significantly lower (*P* < 0.001) in the APT-treated group than the NT group (1.35%, 3/222 cows and 7.3%, 18/245 cows, respectively). No correlation was found between cure and recovery in the NT group, whereas in the APT-treated cows, cure was highly correlated with recovery.

### Changes in individual cows’ milk yield and in total volume milked into the bulk milk tank

**Fig 3:**
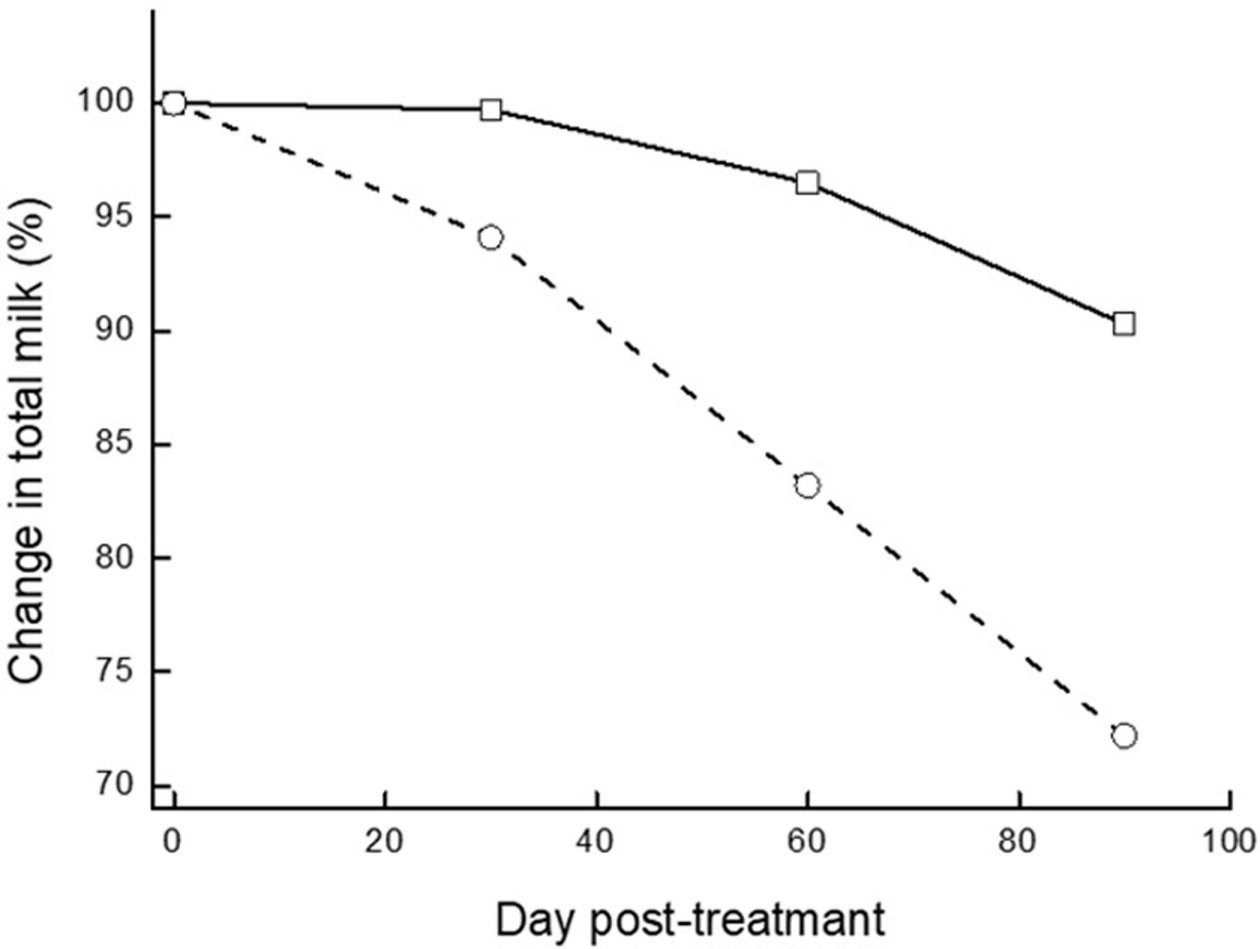
The effect of APT treatment on the changes in the volume of milk in the bulk milk tank (%) during the first 90 d post-treatment of cows with subclinical mastitis event. Changes in total bulk milk (%) during the first 90 d post-treatment of cows with subclinical mastitis event (SCC >1 × 10^6^ cell/mL); cows were treated with acoustic pulse technology (APT; □, n = 222), no treatment (NT; ◯, n = 245). The MY of each group on day of treatment was set at 100% and changes are presented as percent from time 0 level.

Changes in individual cows’ MY were calculated as post-treatment test-day MY relative to that at treatment (time 0), the latter set to 100%; thus, MY was expressed as Δ% of the volume at time 0. The average changes within each of the 2 groups were significant (*P* < 0.05). The mean of the individual MY in the APT-treatment group increased by 3.8% on d 30, decreased by 0.6% on d 60 and decreased by 4.5% on d 90 post-treatment. In the NT group, mean individual MY decreased at all-time points from 3.0% on d 30 to 16.6% and 26.4% on d 60 and 90, respectively. As a result of the changes in MY and cow culling, the volume of milk in the bulk milk tank changed (Fig 3). The changes in the milked volume were calculated as total bulk milk in the tank at the post-treatment milk recordings relative to that at treatment (time 0), which was set to 100%; changes were expressed as Δ% of the volume at time 0. During 90 d post-treatment, the average volume of milk per cow in the APT-treated group increased by 0.47% at 30 d and then decreased by 2.2% at 60 d and 6.1% at 90 d; the reduction was significantly less than that in the NT group: 6.3% at 30 d, 16.6% at 60 d and 25.1% at 90 d (Fig 3).

### Economic analysis–Impact of s

An economic cost–benefit analysis was performed for treatment of subclinical mastitis with APT compared to no treatment (Table 3). For the calculation, the average MY of the Israeli herd was 40 L/d or 12,200 L/305 d, where 18% of the cows were identified with subclinical mastitis (first elevation of SCC >1 × 10^6^ cells/mL) (Herd Book of the ICBA). The milk price was fixed at $0.32/L and price for a replacement heifer was fixed at $1,500 (based on ∼1,500 USD [31, 32], and UK £1,700–2,000 [33–35]). According to the current study, the decrease in milk production for NT cows with subclinical mastitis at 90 d was 25.1%, with 16.7% culling rate for these cows. In the APT-treated group, the decrease in milk production reached 9.0% at 90 d and 1.35% culling rate. This means that by treating subclinical cows with APT, one can expect an addition of 16.1% (25.1–9.0) in milk production after treatment and a reduction of 92.8% in culling compared to NT cows.

**Table 3:** Summary of the economic benefits of using acoustic pulse technology (APT) on a dairy farm.

Parameters:						
Cows (#)				100		
% Subclinical mastitis (SCM)				18		
SCM cows (#)				18		
Milk (L/d)				40		
Lactation (d)				305		
Milk (L/305 d)				12,200		
Avg. SCM period (d)				150		
Price per liter ($)				0.32		
Loss per culled cow ($)				1,500		
**NT: Milk reduction**			**Economic gain with APT:**			
Reduction in milk production (%)		25.10	Gain in milk production (%)		16.10	
Milk loss (L/d)		10.04	Daily milk gain (L/d)		6.44	
Total milk loss in 150 days (L)		1,506	Total milk gain in 150 d (L)		966	
Total loss per cow ($)		482	Total gain ($) per cow		309	
Total loss per 100 cows ($)		8,675	Total gain per 100 cows ($)		5,564	
**NT: Culling**			**Economic gain with APT:**			
% Culling of SCM cows		16.70	Reduction in culling (%)		92.80	
Culled SCM cows (#)		3	Cows remaining (#)		2.8	
Total loss from culling ($)		4,509	Total gain from reduced culling ($)		4,185	
**NT: Future milk production**			**Economic gain with APT:**			
Culled SCM cows (#)		3	Cows remaining (#)		2.8	
Milk loss (L /150 d)		18,036	Milk gain (L/150 d)		16,740	
Total milk loss ($)		5,772	Total gain from milk ($)		5,357	
**Total loss per 100 cows ($)**		18,955	**Total gain per 100 cows ($)**		15,106	
**Cost of APT**						
Usage fee ($/cow)						60
Total cost per 100 cows ($)						1,080
**Treatment time with APT**						
Treatment time per cow, h						0.1
Total treatment time for 100 cows, h						18
Cost ($/h)						20
Total time cost per 100 cows ($)						360
**Total cost per 100 cows ($)**						**1,440**

Consequently, average daily MY in cows with subclinical mastitis amounts to 29.95 L/d (25.1% reduction from 40 L/d) in the NT group, compared to 36.39 L/d (9% reduction from 40 L/d) in the APT group. If subclinical mastitis is considered to affect, on average, 150 DIM, there is a total gain of 966 L/infected cow ((36.39–29.95) × 150 d) or $309 (966 × $0.32) gain per APT-treated cow. For a herd of 100 cows, the total gain is $5,564 (18 × $309). In addition, in the study, a 16.7% culling rate was observed in the NT group, compared to 1.35% in the APT group. This means that by treating with APT, the risk for culling of subclinical cows is reduced by over 90%, which adds an extra gain of $4,185 for a herd of 100 animals (2.8 cows × $1,500). The reduction in culling consequently increases the number of cows that remain in the herd for the entire lactation period, giving an additional 16,740 L (2.8 × 40 × 150 d) or $5,357 for a herd of 100 cows. The total economic benefit of using APT on subclinical cows per 100 cows is $15,106. Utilizing APT equipment incurs a cost of $60 per treated cow or $1,080 annually for a 100-cow herd (calculated as 18 cows × $60). The treatment duration for each cow is around 1 hour, resulting in an estimated labor cost of $360 per 100-cow herd per year (18 cows × $20/h). The total additional expenses, including APT equipment usage and treatment time, amounts to $1,440 for every 100 cows. Notably, the return on investment is $10.5 for each $1 invested in APT when applied to the treatment of subclinical cows ($15,106 / $1,440).

## Discussion

We determined the percentage of recovery rather than IMI cure rate of cows identified with mastitis. Successful cure of IMI is related to the elimination of a pathogen with time which is undoubtedly important in the healing process. However, cure is only the first step to recovery. Thus, in most cases (except for highly infectious pathogens such as *Streptococcus agalactiae*, *Staphylococcus aureus* etc.), the economic burden stems from the inflammation, which negatively influences MY and quality. We performed a bacteriological analysis for two of the studied farms. The distribution of the bacteria before treatment among the 2 treatment groups was similar, with ∼25% Gram positive and ∼50% Gram negative. The high level of *Escherichia coli* isolated from cows with no clinical symptoms was unusual, because *E*. *coli* is a major pathogen in acute mastitis [36–39] and, together with its high persistence for weeks, we suggest that this strain is probably less violent but more persistent in the mammary gland.

Cure from the bacteria was calculated only for cows from which bacteria were isolated in the pretreatment and not for NBF cows, as suggested by Oliveira et al. [40]. The percent of spontaneous (with no treatment) bacterial cure (NT group) was 47.4%, thus >50% of the NT cows developed chronic infection, in line with other studies [41]. It is important to mention that: 1) both samplings, before and after treatment were only performed once; and 2) bacteriological status was not a criterion for the selection and the distribution of the pathogens were not equal between the groups, the results should therefore be treated with caution. In light of the limitations regarding bacteriology, in this article: retrospective evaluation of udder recovery, an additional study was conducted focusing on assessing bacterial cure vs. recovery from the inflammation [42]. The results of that study showed 65–75% recovery of cows with first time SCC elevation, however with low correlation between them. Moreover, in chronic subclinical mastitis both the cure and recovery rates were significantly low.

In cases of subclinical mastitis, which on many farms are not noted, the practice is to ignore it regardless of SCC level, and only to dry off the gland or treat the animal with antibiotics in severe cases. Thus, subclinical mastitis contributes to economic losses, because high SCC is usually associated with lower MY and decreased milk quality in the bulk milk tank [43–46]. Moreover, this milk is unsuitable for human consumption and thus forces culling of the affected cow [47]. Introduction of APT as a novel treatment option for those cows during lactation resulted in 81.3% bacterial cure—1.6 times higher than the spontaneous cure in the NT group. However, as already noted, the key target is recovery from inflammation, not bacterial cure. Indeed, the 47.4% spontaneous cure in the NT cows was significantly higher than the 11.5% recovered cows in that group; thus, no correlation was found between cure and recovery. This indicates that elimination of the bacteria was temporary (only 1 sampling), or that despite their elimination, the gland remained inflamed. In a recent review [39], it was concluded that “associations between bacteriological cure and clinical outcomes are very weak” and “evaluation of continued decline in quarter-level SCC appears to be the most reliable indicator of success.” This conclusion supports the need to determine the recovery from mastitis [1] of a mammary gland and the whole cow according to regained milk production and quality as the main targets. This is done in order to overcome the differences in determining cured udder function, despite the already noted importance of bacterial cure to the healing process. This is crucial because inflammation of the mammary gland, and not only presence of the pathogen, is a key indicator of a disturbance that results in negative changes in milk composition, such as decreased lactose level, increased ion concentration, impaired coagulation properties and increased SCC and its distribution [48, 49]. The lower recovery of heifers ≥ 2 parties is not clear however, most are from the NT group and may suggest lower spontaneous recovery, although further study is necessary to verify this point.

The APT-treated group in this study showed 67.3% recovered cows, similar to our previous results [28–30]. The high correlation between recovery and bacterial cure after APT treatment suggests that the shockwaves promote an immunomodulatory effect, shifting macrophages from M1 (pro-inflammatory activity) to M2 (anti-inflammatory activity) [50–52]; thus, APT not only stimulates tissue healing but also clears the pathogen, due to enhancement of immune system activity.

The low recovery of cows with mastitis (SCC >1 × 10^6^ cells/mL) in the NT group led to forced culling due to reduced MY and high SCC. This reflects the problematic consequences of mastitis, which leads to high culling, especially of primiparous heifers and therefore to loss of their maximum production potential. Studies calculating herd economics, cow longevity and reasons for culling [53–57] have shown that at least 50% of all culls are primarily declared to be health-related. Moreover, in a review by Beaudeau et al. [58], mastitis was found to carry the most direct culling risk.

The APT treatment closes the gap between the demand for a solution to treat mastitis that addresses growing consumer and public health authority concerns about AMR, biosecurity and animal welfare issues and the need to alleviate the economic consequences of mastitis for the farmer. Policymakers are restricting the use of antibiotics in animals to the treatment, control, or prevention of specific diseases. In the current study, >90% of the cows treated with APT remained in the herd and depending on the SCC-cutoff level, had higher recovery rates than NT cows, thus enhancing animal welfare and economic balance. Treatment of mastitis by APT significantly increases the cow’s lifetime and significantly reduces the need to introduce new heifers, thus cutting the farmers’ losses.

Subclinical mastitis-associated costs in the current study were $20,550 per herd of 100 NT cows. In a comparable study performed with Canadian herds, it was reported that out of the $52,300/100 cows spent annually, 48% was related to subclinical mastitis, 34% to clinical mastitis and 15% to preventive measures [17]. In the present study, high culling, dried-off glands and the persistence of SCC >1 × 10^6^ cells/mL 90 d post-treatment in the NT group resulted in a sharp reduction in the bulk milk volume. This forced the introduction of replacement heifers and highlights the question of whether broad antibiotic use is actually needed in the dairy industry.

All in all, most of the cows treated with APT remained in the herd, were productive and profitable and continued to produce a high volume of milk. The treatment is not invasive; it is harmless to the animal and has no side effects. The cows were milked during treatment into the bulk milk tank, thus avoiding discarded milk; it did not affect the milking routine and it is environmentally friendly. Above all, milk was free of antimicrobial pressure that might cause the development of resistant bacteria, thus addressing welfare issues.

## Conclusions

The analysis compared recovery from inflammation, culling and milk yield of cows with subclinical mastitis that were treated with APT. The percent recovery of cows treated by APT was >75%, significantly higher (*P <* 0.001; ∼7-fold) than the NT group. Cure from bacteria in the NT group was 32.7% and in the APT-treated cows it was 83.9%, which highly correlated with recovery. It is important to mention that both samplings, before and after treatment were only performed once, and bacteriological status was not a criterion for the selection, thus the results should be treated with caution. During the 90 d post-treatment, culling due to a mastitis event was significantly different (*P* < 0.001) with only 1.35% of the cows in the APT group and 16.7% cows in the NT group. As a result, 98.8% of the APT-treated group remained in the herds compared to 84.1% of the NT group (*P* < 0.001). During the 90 d of the study, the changes in individual cows’ MY was significantly different (*P* < 0.05), lower in the APT group. As a result, the volume of milk in the bulk milk tank owing to the changes in MY and cow culling at 90 d post-treatment, were 6.1% at the APT group compared to 25.1% in the NT group. All in all, the APT treatment resulted in increased income due to higher recovery, higher milk production, and decreased costs for unnecessary antibiotic treatment, and discarded involuntary culling. The total economic benefit of using APT on subclinical cows per 100 cows exceeded $15,000.

Further data collection from commercial users will establish the full cost-benefit of this new technology.

## Supporting information

S1 File (XLSX)
